# Sporadic Case of Visceral Leishmaniasis in Sikkim, India

**DOI:** 10.4103/0974-777X.62865

**Published:** 2010

**Authors:** Luna Adhikari, T S K Singh, Dechenla Tsering, O P Dhakal, Amlan Gupta

**Affiliations:** *Department of Microbiology, Sikkim-Manipal Institute of Medical Sciences, 5^th^ Mile Tadong, Gangtok, Sikkim - 737 102, India*; 1 *Department of Community Medicine, Sikkim-Manipal Institute of Medical Sciences, 5^th^ Mile Tadong, Gangtok, Sikkim - 737 102, India*; 2 *Department of Medicine, Sikkim-Manipal Institute of Medical Sciences, 5^th^ Mile Tadong, Gangtok, Sikkim - 737 102, India*; 3 *Department of Pathology, Sikkim-Manipal Institute of Medical Sciences, 5^th^ Mile Tadong, Gangtok, Sikkim - 737 102, India*

Sir,

Leishmaniasis, an arthropod–borne disease is caused by *Leishmania donovani* complex transmitted by Phlebotomus sandflies. In India, visceral leishmaniasis has been known to occur in epidemics and endemics in eastern parts of the country, West Bengal, Eastern Districts of Uttar Pradesh, Assam and foothills of Sikkim,[1,2] In recent years, many development projects have come up in Sikkim especially in the foothills. This has led to the immigration of laborers from the nearby states like Bihar and West Bengal, which are known endemic regions. In addition, these development projects expose more virgin population and bring human beings into areas of vector and reservoir concentration.[3] This report describes a visceral leishmaniasis (VL) case initially diagnosed as chronic liver disease. A 50-year-old male farmer from Namchi, South Sikkim, presented with a history of intermittent fever associated with chills and rigor since one year, abdominal distension and swelling of feet with intermittent epistaxis for the last six months. There was no history of blood transfusion, accidental needle stick injuries and travel to any known endemic area. The patient was treated earlier for chronic liver disease with decompensation. Physical examination revealed pallor, moderate splenomegaly (firm and non tender) and ascites. There was no icterus, no skin pigmentation, no weight loss, no lymphadenopathy, no hepatomegaly and no loss of hair. All the vital signs were within normal limits. Routine hematological investigation showed leucopenia (2000/mm^3^), hemoglobin – 6.8 gm/dl, platelet count – 74000/mm^3^ (thrombocytopenia). Liver function tests and blood sugar (random) was also within normal limits. Aldehyde test was positive. Ultra sonography showed gross splenomegaly (size 19 cm) with minimal ascites and normal liver echotexture. Endoscopy revealed grade I – II esophageal varices with portal gastropathy. A diagnostic bone marrow aspirate and splenic puncture were performed. Giemsa stained smears of splenic aspirate [Figure 1] and bone marrow showed numerous LD bodies within histocytes and also extracellular LD bodies. Parasite grading was done according to WHO recommendation on the estimation of *L. donovani* amastigote numbers in splenic aspirate smear.[^6^] Pre-treatment parasite count was 4+ (251 LD bodies/100 OIF (Oil immersion field). Bone marrow smear showed granulocyte series within normal limits but lymphoid series was markedly increased without abnormal aggregates and devoid of granulomas. There were a few granulomas in the splenic aspirate [Figure 2]. The case was diagnosed as visceral leishmaniasis and was treated with sodium Stibogluconate (20 mg/kg IM (intramuscular) daily).

**Figure 1:**
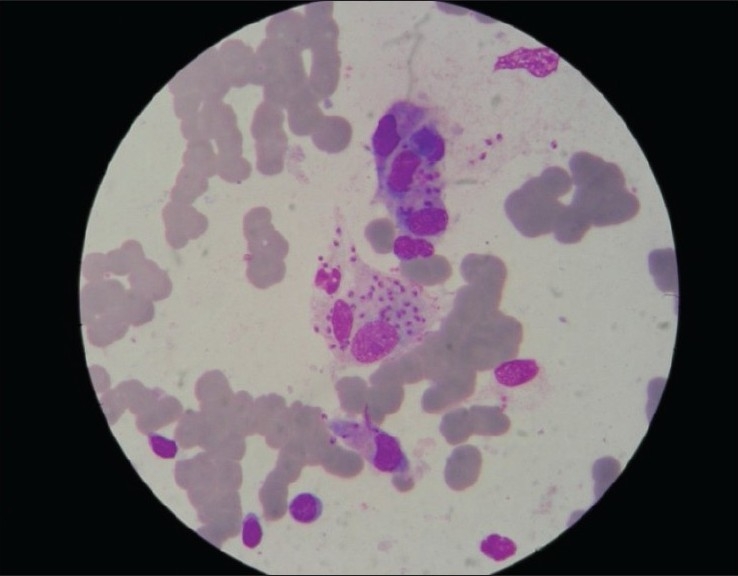
Histopathology of splenic aspirate showing granulomas

**Figure 2:**
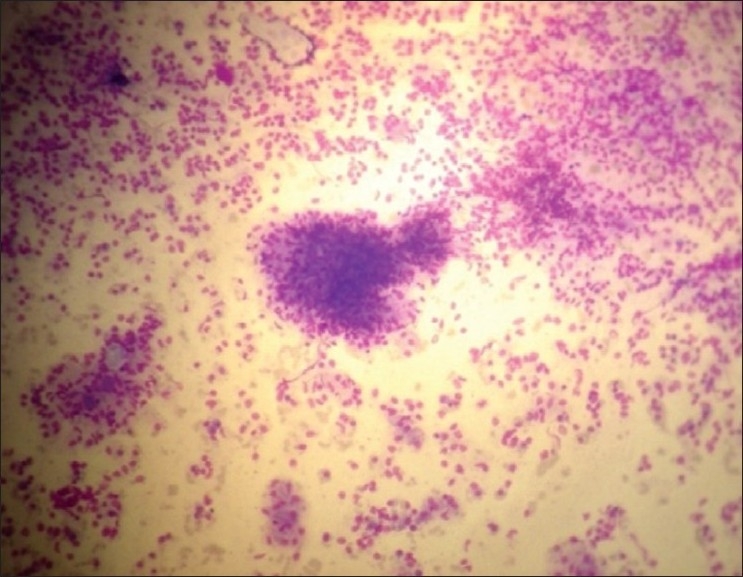
*Leishmania donovani* bodies in splenic aspirate

Repeat splenic aspirate was made three days after sodium Stibogluconate therapy to evaluate the response of the patient to the therapy. The count of LD bodies reduced to 3+ (26 LD bodies/100 OIF) and showed some morphological changes. We are reporting this case because of atypical clinical presentation, undetermined mode of transmission and as the prevalence of the disease is not known in Sikkim. In conclusion, in cases of visceral organ pathology accompanied by persistent fever and hematological disorders, parasitic infections, particularly visceral leishmaniasis should be considered in the differential diagnosis.[4]

## References

[1] Mahajan RC, Mohan K, Ozcel MA, Alkan MZ (1996). Epidemiology of visceral leishmaniasis and its control. Parasitology for the 21st Century.

[2] Marinkelle CJ (1980). The control of leishmaniases. Bull World Health Organ.

[3] Park K Park's Textbook of Preventive and Social Medicine.

[4] Yazici P, Yenιay L, Aydin U, Taşbakan M, Ozütemιz O, Yilmaz R (2008). Visceral leishmaniasis as a rare cause of granulomatosis hepatitis: A case report. Turkiye Parazitol Derg.

